# Tripterygium Glycosides Extract-Induced Hepatic Cholestasis: A Mechanistic Study Using a Microfluidic Liver-on-a-Chip System

**DOI:** 10.3390/ijms27094154

**Published:** 2026-05-06

**Authors:** Yifei Yang, Ya Zhang, Yun Yang, Bing Xia, Haijing Zhang, Guozhuang Zhang, Ping Gong, Ying Qi, Zhe Wu, Chun Li, Ting Liu

**Affiliations:** Institute of Chinese Materia Medica, China Academy of Chinese Medical Sciences, State Key Laboratory for Quality Ensurance and Sustainable Use of Dao-di Herbs, Beijing 100700, China; yfyang@icmm.ac.cn (Y.Y.); zhangya190726@163.com (Y.Z.); leyla20000219@163.com (Y.Y.); bxia@icmm.ac.cn (B.X.); hjzhang@icmm.ac.cn (H.Z.); gzzhang@icmm.ac.cn (G.Z.); pgong@icmm.ac.cn (P.G.); qidabao0225@163.com (Y.Q.); zwu@icmm.ac.cn (Z.W.); cli@icmm.ac.cn (C.L.)

**Keywords:** precision-cut liver slices, bile acid transporters, oxidative stress, farnesoid X receptor, drug-induced liver injury

## Abstract

Tripterygium glycosides extract (TGE), the primary active component of tripterygium glycosides tablets, is widely used for immune-related disorders but raises significant clinical concerns regarding cholestatic drug-induced liver injury. As conventional models fail to fully recapitulate the complex pathogenesis of traditional Chinese medicine toxicity, this study aimed to elucidate the mechanisms of TGE-induced cholestatic injury using a biomimetic microfluidic liver-on-a-chip platform. The chip integrated rat precision-cut liver slices (PCLSs) and human endothelial cells (EA.hy926) to simulate the hepatic sinusoidal microenvironment. Following TGE exposure (15–135 μg/mL for 12 and 24 h), vascular barrier integrity was maintained, while liver injury markers (ALT, AST, TBA, DBIL) significantly increased in a dose- and time-dependent manner, accompanied by progressive histopathological deterioration in PCLSs. Mechanistically, TGE triggered severe oxidative stress (decreased SOD/GSH/GSH-Px and increased MDA) and upregulated pro-inflammatory cytokines (IL-4 and IL-1β). Consequently, the expression of the bile acid receptor FXR and transporters (BSEP and MRP2) was significantly downregulated. In conclusion, TGE induces cholestatic liver injury via a sequential pathway: oxidative stress initiates an immune-inflammatory response, which subsequently suppresses the FXR/BSEP/MRP2 axis. Future studies should focus on developing fully humanized liver-on-a-chip systems to further validate these mechanisms and improve clinical translational significance.

## 1. Introduction

Tripterygium glycosides tablets (TGTs), known as “Chinese herbal hormones”, have been widely used in the treatment of rheumatoid arthritis, autoimmune hepatitis, and other immune-related diseases owing to their immunosuppressive and anti-inflammatory effects [1,2]. The main ingredient of TGTs is tripterygium glycosides extract (TGE), a lipid-soluble mixture refined from the roots of the plant *Tripterygium wilfordii* Hook F. With TGE being widely applied in clinical practice, its safety has increasingly attracted scholarly and regulatory attention [3,4]. Data from the Adverse Drug Reaction (ADR) Case Report Database of the National Center for Adverse Drug Reaction Monitoring revealed that *T. wilfordii* preparations are associated with hepatotoxicity, nephrotoxicity, hematotoxicity, and reproductive toxicity. According to the ADR report, between 2004 and September 2011, a total of 633 ADR case reports involving TGT were documented; among these, 53 cases (8.4%) were classified as serious, with the most common manifestations including drug-induced hepatitis, acute kidney injury, granulocytopenia, leukopenia, thrombocytopenia, amenorrhea, oligospermia, and cardiac arrhythmias (https://www.nmpa.gov.cn/xxgk/yjjsh/ypblfytb/20120401101301366.html accessed on 11 March 2026) [5]. Most patients exhibited non-specific prodromal symptoms, including anorexia, nausea, and fatigue. Clinical manifestations concurrently include hepatomegaly, elevated serum levels of alanine aminotransferase (ALT) and aspartate aminotransferase (AST), and notable cholestasis [6].

TGE or its main toxic component triptolide (TP) can cause definite cholestatic liver injury, involving multiple injury mechanisms such as lipid peroxidation, apoptosis and autophagy, immune-mediated damage, and drug-metabolizing enzyme activities [7,8,9,10,11]. Nevertheless, the precise molecular mechanism underlying TGE-induced cholestatic hepatotoxicity remain unclear and requires further in-depth investigation.

The liver-on-a-chip model is a microfluidic device designed to mimic the physiological and biochemical characteristics of the liver in vitro. Microfabrication techniques enable the generation of highly refined and complex channel architectures on a chip by integrating hepatocytes or liver tissue constructs with microfluidic technology. These microchannel networks give rise to biomimetic tissue structures and microenvironments at the micrometer scale. Currently, microfluidic chips can simulate complex in vivo-like microenvironments through the strategic integration of microchannels, reaction chambers, and functional detection components, thereby enabling precise control over the cellular or tissue culture microenvironment [12].

Conventional hepatic cell lines (e.g., HepG2) and animal models are increasingly recognized as insufficient for elucidating the mechanisms of complex drug-induced liver injury (DILI). Monoculture systems often lack crucial hepatocyte–non-parenchymal cell crosstalk and adequate drug-metabolizing enzyme expression, leading to a poor prediction of clinical hepatotoxicity, especially for complex mixtures such as herbal medicines [13,14]. To overcome these limitations, liver-on-a-chip platforms have emerged as superior tools. However, mimicking the complex metabolic processing and hepatic microenvironment in the context of TGE remains a significant challenge. We therefore engineered a customized microfluidic liver-on-a-chip system, designed to optimally model the multifaceted pathogenesis of TGE-induced cholestasis. First, to accurately replicate the in vivo metabolic capacity necessary for bioactivating complex components such as TGE, we utilized rat precision-cut liver slices (PCLSs). Unlike simplified cell lines, PCLSs preserve the complete native tissue architecture, including the full repertoire of liver-resident immune cells and primary drug-metabolizing enzymes which are crucial for probing immune-mediated damage and oxidative stress. Second, to simulate the hepatic sinusoidal barrier—which plays a vital role in maintaining cholestasis homeostasis—we integrated a monolayer of EA.hy926 cells to form biomimetic blood vessels. This specific configuration synergistically bridges the gap between simplistic monocultures and whole-animal models. By faithfully recapitulating this intricate hepatic sinusoidal microenvironment, our tailored chip system provides a highly predictive tool for mechanistically exploring TGE-induced cholestatic liver injury.

## 2. Results

### 2.1. Effect of TGE on the Viability of EA.hy926 Cells and HepG2 Cells

**Figure 1:**
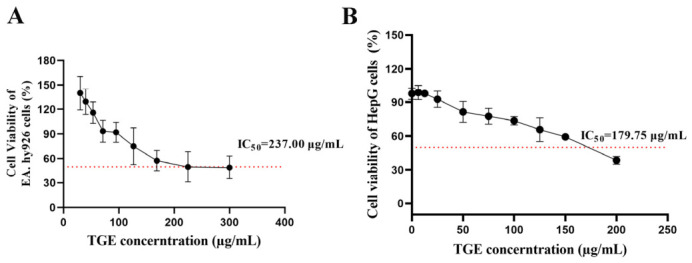
Effect of TGE on the viability of EA.hy 926 cells and HepG2 cells. (**A**) Viability of EA.hy926 cells following treatment with indicated concentrations of TGE. (**B**) Viability of HepG2 cells following treatment with indicated concentrations of TGE. The red dotted line indicated the 50% cell viability level.

Figure 1 shows that TGE suppressed HepG2 cell proliferation in a concentration-dependent manner, with higher concentrations eliciting progressively greater inhibitory effects. The half-maximal inhibitory concentration (IC_50_) of TGE was 179.75 μg/mL for HepG2 cells (Figure 1B) and 237.00 μg/mL for EA.hy926 cells (Figure 1A). Based on the dose–response relationship observed in HepG2 cells, three sub-cytotoxic concentrations—15 μg/mL (TGE-L), 45 μg/mL (TGE-M), and 135 μg/mL (TGE-H)—were selected for subsequent functional assays, corresponding to approximate inhibition rates of 3–4%, 10–15%, and 40–45% in HepG2 cells, respectively.

### 2.2. Effect of TGE on the Morphology of EA.hy926 Cells in the Microfluidic Liver-on-a-Chip System

**Figure 2:**
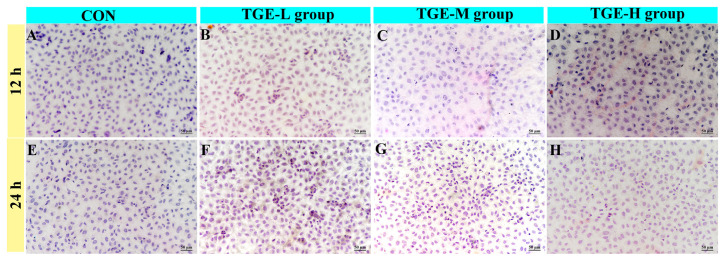
Effect of TGE on the morphology of EA.hy926 cells. Representative phase-contrast images showing EA.hy926 cell morphology after 12 and 24 h of culture in the microfluidic liver-on-a-chip system. (**A**) Control group at 12 h. (**B**–**D**) TGE-L, TGE-M, and TGE-H groups, respectively, at 12 h. (**E**) Control group at 24 h. (**F**–**H**) TGE-L, TGE-M, and TGE-H groups, respectively, at 24 h.

Figure 2 shows that EA.hy926 cells cultured in the microfluidic liver-on-a-chip system for 12 and 24 h post-drug administration exhibited no significant morphological alterations across all TGE-treated groups compared with the control group. The cells maintained a spindle-shaped morphology, were densely and uniformly aligned, displayed well-defined cellular edges and sharp nuclear–cytoplasmic boundaries, and retained a normal nuclear-to-cytoplasmic ratio.

### 2.3. Effect of TGE on the Expressions of VE-Cadherin and ZO-1 in EA.hy926 Cells

**Figure 3:**
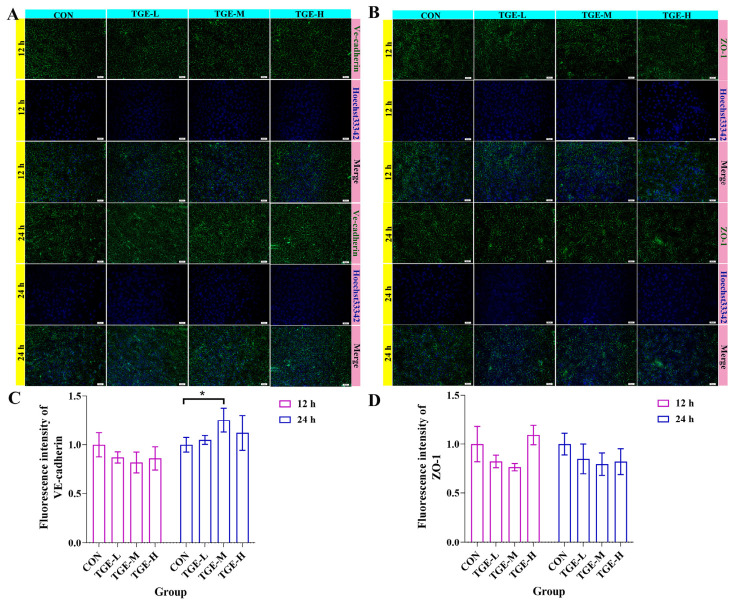
Effect of TGE on the expression of VE-cadherin and ZO-1 in EA.hy926 cells. Representative immunofluorescence images showing the expression and localization of endothelial junction proteins in EA.hy926 cells after 12 and 24 h of culture in the microfluidic liver-on-a-chip system. (**A**) VE-cadherin expression (green) in control and TGE-treated groups at 12 and 24 h. (**B**) ZO-1 expression (green) in control and TGE-treated groups at 12 and 24 h. Nuclei were counterstained with Hoechst 33342 (blue). (**C**) Fluorescence intensity of VE-cadherin. Culture for 12 h: TGE-L vs. CON, *p* = 0.3545; TGE-M vs. CON, *p* = 0.1541; TGE-H vs. CON, *p* = 0.3084. Culture for 24 h: TGE-L vs. CON, *p* = 0.8829; TGE-M vs. CON, *p* = 0.0262; TGE-H vs. CON, *p* = 0.3582. (**D**) Fluorescence intensity of ZO-1. Culture for 12 h: TGE-L vs. CON, *p* = 0.1733; TGE-M vs. CON, *p* = 0.0626; TGE-H vs. CON, *p* = 0.5624. Culture for 24 h: TGE-L vs. CON, *p* = 0.2728; TGE-M vs. CON, *p* = 0.1061; TGE-H vs. CON, *p* = 0.1682. * *p* < 0.05 compared with the control group. *n* = 3 or 4 (data are shown in Supplementary File S2).

Immunofluorescence staining of EA.hy926 cells confirmed robust membrane-localized expression of the endothelial junction proteins VE-cadherin and ZO-1. At 12 h post-TGE administration, no significant changes in the fluorescence intensity or membrane distribution of either protein were observed across all treatment groups compared with the control (Figure 3A–D). By 24 h, however, VE-cadherin signal intensity was elevated in the TGE-M group (*p* = 0.0262; Figure 3A,C), whereas ZO-1 expression remained unchanged (Figure 3B,D). These findings indicate that TGE—within the tested concentration range—does not clearly disrupt endothelial intercellular junction integrity.

### 2.4. Influence of TGE on the Levels of Liver Injury Markers in Bionic Blood of the Microfluidic Liver Organ-on-a-Chip System

Table 1 reveals that following 12 h of drug administration and culture within the microfluidic liver-on-a-chip system, no statistically significant difference was observed in the levels of aspartate AST, mAST, ALT, LDH, GGT, TBA or DBIL between any of the TGE-treated groups and the control group at this time point.

After 24 h of treatment, more pronounced alterations were evident. Significant increases in AST, mAST, LDH, and TBA concentrations were observed in the TGE-M group relative to controls (*p* = 0.0159, *p* = 0.0064, *p* = 0.0004, and *p* = 0.0429, respectively). The TGE-H group exhibited statistically significant elevations in ALT and DBIL levels compared to the control group (*p* = 0.0345 and *p* = 0.0082, respectively). Notably, the increase in DBIL exhibited a clear dose-dependent pattern.

### 2.5. Effect of TGE on the Histological Structure of PCLSs in the Microfluidic Liver-on-a-Chip System

**Figure 4:**
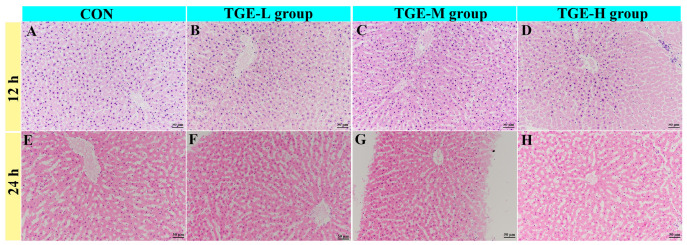
Effect of TGE on the histological structure of PCLSs in the microfluidic liver organ-on-a-chip system. Representative hematoxylin and eosin (**E**,**H**)-stained sections of PCLSs after 12 and 24 h of culture. (**A**) Control group at 12 h. (**B**–**D**) TGE-L, TGE-M, and TGE-H groups, respectively, at 12 h. (**E**) Control group at 24 h. (**F**–**H**) TGE-L, TGE-M, and TGE-H groups, respectively, at 24 h.

Histological examination revealed time- and concentration-dependent morphological alterations in PCLSs following TGE treatment (Figure 4 and Table 2). After 12 h of culture, mild focal hepatocyte swelling was observed in the TGE-L group (incidence rate 2/3, similarly hereinafter, Figure 4B). This effect was more pronounced in the TGE-M group, which exhibited focal to moderate hepatocyte swelling (3/3, Figure 4C), while the TGE-H group showed moderate hepatocyte swelling (3/3) accompanied by occasional karyolysis (3/3, *p* < 0.05, Figure 4D). Following 24 h of treatment, mild to moderate hepatic sinusoidal dilation was evident even in the control group (1/3, Figure 4E). In the TGE-L group, additional changes included mild to moderate hepatocyte degeneration, swelling, and edema (3/3, Figure 4F). The TGE-M group displayed more severe pathology, and multifocal karyolysis superimposed on the lesions was observed at the lower dose (3/3, Figure 4G). In the TGE-H group, diffuse sinusoidal dilation, hepatocyte swelling, and karyolysis were present after 24 h (3/3, *p* < 0.05, Figure 4H). The consistent increase in histological scores from 12 h to 24 h across all TGE groups indicated a biologically meaningful, time-dependent progression of injury (Table 2). These quantitative data support the qualitative observation that TGE induces hepatocellular injury in a time- and concentration-dependent manner, with the most pronounced effects observed in the TGE-H group at 12 h and in the TGE-M/H groups at 24 h. However, larger sample sizes are needed to confirm the statistical significance of these differences. Individual animal semi-quantitative histological scores for all experimental groups were shown in the Supplementary Files Table S1.

### 2.6. TGE Induced Oxidative Stress in PCLSs in the Microfluidic Liver-on-a-Chip System

**Figure 5:**
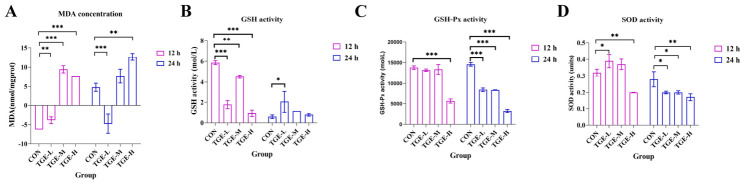
Effect of TGE on oxidative stress-related indicators in PCLSs within the microfluidic liver organ-on-a-chip system. PCLSs were collected after 12 and 24 h of TGE treatment for biochemical analysis. (**A**) MDA levels. Culture for 12 h: TGE-L vs. CON, *p* = 0.0046; TGE-M vs. CON, *p* < 0.0001; TGE-H vs. CON, *p* < 0.0001. Culture for 24 h: TGE-L vs. CON, *p* = 0.0003; TGE-M vs. CON, *p* = 0.1536; TGE-H vs. CON, *p* = 0.0011. (**B**) GSH content. Culture for 12 h: TGE-L vs. CON, *p* < 0.0001; TGE-M vs. CON, *p* = 0.0011; TGE-H vs. CON, *p* < 0.0001. Culture for 24 h: TGE-L vs. CON, *p* = 0.0217; TGE-M vs. CON, *p* = 0.4487; TGE-H vs. CON, *p* = 0.9391. (**C**) GSH-Px activity. Culture for 12 h: TGE-L vs. CON, *p* = 0.5735; TGE-M vs. CON, *p* = 0.7819; TGE-H vs. CON, *p* < 0.0001. Culture for 24 h: TGE-L vs. CON, *p* < 0.0001; TGE-M vs. CON, *p* < 0.0001; TGE-H vs. CON, *p* < 0.0001. (**D**) SOD activity. Culture for 12 h: TGE-L vs. CON, *p* = 0.0346; TGE-M vs. CON, *p* = 0.1300; TGE-H vs. CON, *p* = 0.0017. Culture for 24 h: TGE-L vs. CON, *p* = 0.0147; TGE-M vs. CON, *p* = 0.0147; TGE-H vs. CON, *p* = 0.0023. Data are presented as mean ± SD (*n* = 3). * *p* < 0.05, ** *p* < 0.01, *** *p* < 0.001 compared with the corresponding control group.

As shown in Figure 5, TGE treatment induced oxidative stress in PCLSs in a time- and concentration-dependent manner. After 12 h of exposure, MDA levels were significantly elevated in all TGE-treated groups compared to the control (*p* < 0.01 and *p* < 0.001; Figure 5A), while the GSH content correspondingly decreased (*p* < 0.01 and *p* < 0.001; Figure 5B). A similar trend of significant decline was noted for GSH-Px activity (TGE-H group) and for SOD activity (TGE-L and TGE-H groups) (*p* < 0.001, *p* < 0.05 and *p* < 0.01, respectively; Figure 5C,D).

Following 24 h of treatment, MDA levels remained significantly elevated in the TGE-H group relative to the control (*p* < 0.01; Figure 5A). A significant increase in the GSH content was observed in the TGE-L group (*p* < 0.05; Figure 5B), while GSH-Px activity was markedly suppressed across all TGE-treated groups (*p* < 0.001; Figure 5C). SOD activity declined in all TGE-treated groups (*p* < 0.05 and *p* < 0.01; Figure 5D).

### 2.7. TGE Induced Inflammation in PCLSs in the Microfluidic Liver-on-a-Chip System

**Figure 6:**
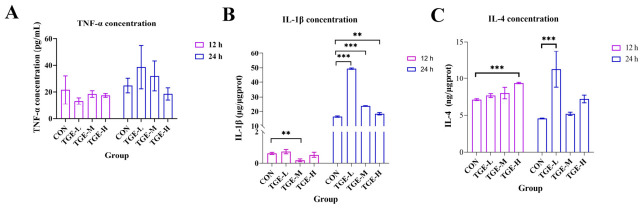
The influence of TGE on immune cytokine levels in PCLSs within the microfluidic liver organ-on-a-chip system. PCLSs were collected after 12 and 24 h of TGE treatment, and cytokine concentrations in tissue homogenates were measured by ELISA. (**A**) TNF-α levels. Culture for 12 h: TGE-L vs. CON, *p* = 0.2353; TGE-M vs. CON, *p* = 0.8372; TGE-H vs. CON, *p* = 0.7074. Culture for 24 h: TGE-L vs. CON, *p* = 0.3063; TGE-M vs. CON, *p* = 0.7357; TGE-H vs. CON, *p* = 0.8200. (**B**) IL-1β levels. Culture for 12 h: TGE-L vs. CON, *p* = 0.5858; TGE-M vs. CON, *p* = 0.0044; TGE-H vs. CON, *p* = 0.6450. Culture for 24 h: TGE-L vs. CON, *p* < 0.0001; TGE-M vs. CON, *p* < 0.0001; TGE-H vs. CON, *p* = 0.0053. (**C**) IL-4 levels. Culture for 12 h: TGE-L vs. CON, *p* = 0.3168; TGE-M vs. CON, *p* = 0.0738; TGE-H vs. CON, *p* = 0.0004. Culture for 24 h: TGE-L vs. CON, *p* = 0.0005; TGE-M vs. CON, *p* = 0.8743; TGE-H vs. CON, *p* = 0.0747. Data are presented as mean ± SD (*n* = 3). ** *p* < 0.01, *** *p* < 0.001 compared with the corresponding control group.

Figure 6 shows that the level of IL-1β in the TGE-M group was lower than in the CON group (*p* < 0.01; Figure 6B) after 12 h of exposure. Meanwhile, in the TGE-H group, IL-4 levels were notably higher than in the CON group (*p* < 0.001; Figure 6C). However, following 24 h of exposure, a marked increase in IL-1β levels was detected across all TGE-treated groups (*p* < 0.001; Figure 6B). In addition, the TGE-L group exhibited significantly elevated IL-4 levels at this time point (*p* < 0.001; Figure 6C). No statistically significant changes in TNF-α levels were observed at either time point (Figure 6A).

### 2.8. Effect of TGE on the Expression of Bile Acid Transport-Related Proteins

**Figure 7:**
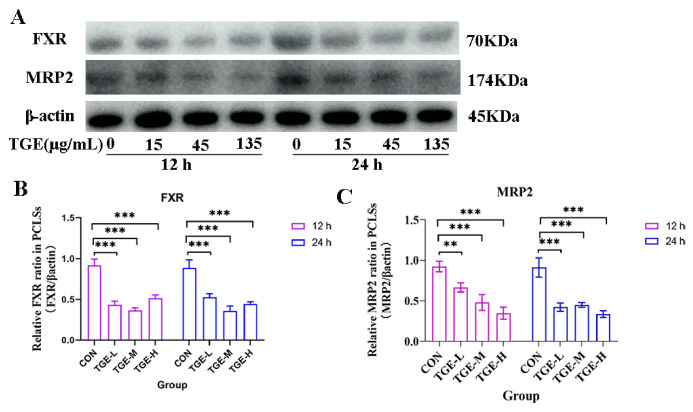
Effect of TGE on the expression of FXR and MRP2 in PCLSs within the microfluidic liver-on-a-chip system. PCLSs were harvested after 12 and 24 h of TGE treatment for Western blot analysis. (**A**) Representative immunoblots showing FXR and MRP2 protein levels; β-actin served as a loading control. (**B**) Densitometric quantification of FXR expression normalized to β-actin. Culture for 12 h: TGE-L vs. CON, *p* < 0.0001; TGE-M vs. CON, *p* < 0.0001; TGE-H vs. CON, *p* < 0.0001. Culture for 24 h: TGE-L vs. CON, *p* = 0.0004; TGE-M vs. CON, *p* < 0.0001; TGE-H vs. CON, *p* < 0.0001. (**C**) Densitometric quantification of MRP2 expression normalized to β-actin. Culture for 12 h: TGE-L vs. CON, *p* = 0.0076; TGE-M vs. CON, *p* = 0.0003; TGE-H vs. CON, *p* < 0.0001. Culture for 24 h: TGE-L vs. CON, *p* < 0.0001; TGE-M vs. CON, *p* = 0.0001; TGE-H vs. CON, *p* < 0.0001. Data are presented as mean ± SD (*n* = 3). ** *p* < 0.01, *** *p* < 0.001 compared with the corresponding control group.

Western blot analysis revealed that TGE treatment modulated the expression of key bile acid transporters in a time- and concentration-dependent manner (Figure 7). At 12 h post treatment, a significant decrease in the protein expression levels of FXR and MRP2 was observed in all three treatment groups (TGE-L, TGE-M, and TGE-H) compared with the control group. This downregulation persisted at 24 h post treatment, with all three doses still showing significantly lower expression levels of both FXR and MRP2 relative to the control group (*p* < 0.01 and *p* < 0.001; Figure 7A–C).

**Figure 8:**
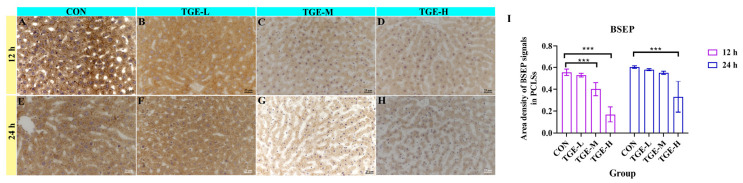
Effect of TGE on the expression of BSEP in PCLSs within the microfluidic liver organ-on-a-chip system. Representative IHC staining of BSEP in PCLSs after 12 and 24 h of TGE treatment. (**A**) Control group at 12 h. (**B**–**D**) TGE-L, TGE-M, and TGE-H groups, respectively, at 12 h. (**E**) Control group at 24 h. (**F**–**H**) TGE-L, TGE-M, and TGE-H groups, respectively, at 24 h. Positive BSEP immunoreactivity appears as granular brown deposits within hepatocytes. (**I**) Semi-quantitative analysis of BSEP expression expressed as area density of positive signals per field of view. Culture for 12 h: TGE-L vs. CON, *p* = 0.7442; TGE-M vs. CON, *p* = 0.0003; TGE-H vs. CON, *p* < 0.0001. Culture for 24 h: TGE-L vs. CON, *p* < 0.9191; TGE-M vs. CON, *p* = 0.5522; TGE-H vs. CON, *p* < 0.0001. Data are presented as mean ± SD (*n* = 5). *** *p* < 0.001 compared with the corresponding control group.

IHC analysis further demonstrated that TGE significantly affected BSEP protein expression (Figure 8). A significant reduction in BSEP protein levels was observed in the TGE-M and TGE-H groups at 12 h of culture (*p* < 0.001), while this reduction persisted until 24 h in the TGE-H group (*p* < 0.001).

## 3. Discussion

In recent years, the widespread use of herbal medicines, coupled with advances in in-depth pharmacological research, has raised awareness of their potential adverse health effects. A two-year survey conducted across 17 university hospitals in Korea revealed that herbal medicines were implicated in over 72% of DILI cases [15]. Similarly, an increasing incidence of herb-induced DILI has been reported in China, Japan, and India [16,17,18]. Among the various manifestations of DILI, cholestasis—characterized by impaired bile formation, secretion, or excretion—represents a clinically significant form of liver injury. This condition is associated with elevated bile acid levels, oxidative stress, mitochondrial dysfunction, and inflammatory damage. Cholestasis accounts for approximately 30% of all DILI cases [17] and poses substantial challenges in drug development, regulation, and clinical practice. The complexity of herbal medicine constituents and their multifaceted mechanisms of action further complicate both preclinical and clinical evaluations. Therefore, the development of sensitive and reliable hepatotoxicity evaluation models, alongside rigorous preclinical safety studies, is essential for mitigating the risk of DILI.

Previous studies have established that TGE can induce definite cholestatic DILI [19]. In one study population, the incidence of liver injury among TGE users was 16% (110/683), with some patients exhibiting slow progression to severe cholestatic liver injury [6]. Prolonged administration or high doses of TGE have been associated with clinical symptoms such as dry mouth, fatigue, anorexia, and jaundice, often accompanied by elevated serum levels of ALT, AST, TBA, and TBIL [20,21]. Despite these observations, the mechanistic basis of TGE-induced cholestatic liver injury remains incompletely understood.

Recent technological advances, particularly the emergence of organ-on-a-chip (OOC) platforms, have revolutionized the toxicity evaluation of multi-component drugs [12,22]. By recapitulating key aspects of organ function at the microscale, OOC technology offers new avenues for studying drug-induced toxicity. Traditional animal models often fail to accurately predict clinical outcomes due to interspecies differences and limited sensitivity. The compositional complexity of herbal extracts such as TGE further compounds this challenge. Microfluidic organ chips not only reduce experimental duration and circumvent issues related to individual variability, but also enhance the predictive accuracy of clinical responses, thereby providing a more robust and comprehensive framework for toxicity assessment.

In the present study, we integrated rat PCLSs, human umbilical vein endothelial cells (EA.hy926), and microfluidic chip technology to construct a biomimetic hepatic sinusoidal model that replicates the in vivo hepatic microenvironment. PCLSs retain the full complement of liver cell types within their native extracellular matrix and metabolic enzyme context, making them a well-established in vitro model for studying hepatic metabolism, xenobiotic toxicity, and DILI [23,24,25]. Karsten et al. demonstrated the sensitivity of PCLS using various known cholestatic agents, including chlorpromazine, cyclosporine A, and glibenclamide [26]. Another previous work has demonstrated that PCLSs from rats can recapitulate cholestatic responses to drugs such as chlorpromazine, cyclosporine A, and glibenclamide, as evidenced by increased bile acid levels and altered expression of bile acid transporters (BSEP and NTCP) and the nuclear receptor FXR [27]. Accordingly, the PCLS-based liver-on-a-chip platform employed in this study is well-suited for investigating the hepatotoxic mechanisms of TGE, as also supported by recent studies advocating the utility of liver organ chips in toxicity screening [28]. Notably, a key limitation of the current study is the hybrid nature of the constructed system, which combines rat PCLSs with human EA.hy926 endothelial cells. While rat PCLSs were deliberately chosen for their superior metabolic competence, it is important to acknowledge certain limitations arising from interspecies differences, particularly in drug metabolism and inflammatory/cytokine responses. Rats and humans exhibit differences in CYP isoform composition, expression levels, and catalytic activity, especially for major drug-metabolizing isoforms such as CYP1A, CYP2C, CYP2D, and CYP3A [29]. For example, human CYP3A4—which metabolizes approximately 50% of marketed drugs—shows female-predominant expression, whereas its rat ortholog CYP3A2 is male-predominant with distinct substrate selectivity [30]. Additionally, rat CYP2C11 has no direct human ortholog [30]. These isoform-level discrepancies, combined with generally faster drug metabolism in rodents due to higher hepatic CYP content relative to body weight [30], may influence the kinetics of toxic metabolite formation and clearance, and consequently affect the interpretation of dose–response relationships in cross-species configurations. Beyond metabolism, rat and human hepatocytes also show certain differences in cytokine signaling profiles that may be relevant for assessing inflammation-mediated hepatotoxicity. Although rat, mouse, and human hepatocytes all express inducible nitric oxide synthase (iNOS) in response to cytokine stimulation, the magnitude and regulatory mechanisms vary across species [31]. Upon stimulation with TNFα, IL-1β, IFNγ, and LPS, NO production follows a hierarchy of rat > mouse > human hepatocytes. Moreover, rat and mouse hepatocytes respond to single cytokine stimuli (TNFα, IL-1β, or IFNγ alone), whereas human hepatocytes respond only to LPS alone [31]. Thus, while the current cross-species configuration serves as a useful exploratory tool, it may not fully represent a predictive platform for clinical hepatotoxicity assessment. Future efforts toward clinical translation could benefit from the development and validation of fully humanized liver-on-a-chip systems that integrate human primary or iPSC-derived hepatocytes alongside relevant human non-parenchymal cell types within a physiologically relevant microfluidic environment.

In in vitro studies, designing culture medium drug concentrations based on clinical plasma concentrations (typically C_max_) is a critical approach to enhancing the physiological relevance of experimental findings. As TGE is a mixture, we selected its representative component, triptolide (which accounts for 0.008% of TGE), as the surrogate to characterize its concentration. Previous clinical studies have reported that following oral administration of TGE, the C_max_ of triptolide in patients is approximately 10 ng/mL, which corresponds to a TGE concentration of 125 μg/mL. Therefore, we established the in vitro drug concentration gradient based on this value—15 μg/mL (TGE-L), 45 μg/mL (TGE-M), and 135 μg/mL (TGE-H). This gradient encompasses the maximum plasma concentration of TGE, thereby enhancing the clinical translational value of this study [32]. In the present research, the morphology of EA.hy926 cells and the expressions of VE-cadherin and ZO-1 were not significantly disrupted in the TGE-treated groups after 24 h of culture in our microfluidic liver-on-a-chip system. These findings indicate that TGE had no significant effect on the morphology of the artificial vascular epithelium or the expression of tight junction proteins, suggesting that endothelial integrity of the biomimetic blood vessels was not compromised by TGE exposure.

In this study, we confirmed TGE-induced liver injury in the microfluidic liver-on-a-chip system. The levels of ALT, AST, mAST, LDH, TBA, and DBIL were significantly elevated at 24 h in the TGE-treated groups. Changes in ALT, ALP, GGT, and TBIL levels are principal laboratory indicators for assessing the presence and severity of DILI. Serum ALT elevation is often preferred over AST in DILI diagnosis due to its higher sensitivity and specificity for detecting liver injury [33]. LDH, a sensitive but non-specific indicator of liver injury, is widely distributed across tissues and organs [34]. Upon liver damage, ALT, AST, and LDH leak from cells, resulting in increased concentrations in blood or tissue fluids. We observed elevated levels of ALT, AST, and LDH, with ALT exhibiting an increase at 24 h, suggesting that TGE induced liver injury in the microfluidic liver-on-a-chip system. The DBIL level increased significantly in the TGE-H group at 24 h, demonstrating a clear dose- and time-dependent relationship. Elevated TBA and DBIL levels are associated with biliary obstruction and may indicate impaired hepatic excretory function [35,36]. These results suggest that TGE treatment could lead to bile acid excretion dysfunction, thereby inducing cholestatic liver injury in the microfluidic liver-on-a-chip system. Moreover, hepatocytes within PCLSs from TGE-treated groups exhibited varying degrees and extents of edema, degeneration, and karyolysis, with these pathological changes displaying a distinct dose- and time-dependent relationship. The histopathological alterations were consistent with the changes observed in liver injury-related biochemical indicators. Notably, a consistent trend was observed across all experimental groups, in which histological scores at 24 h were uniformly higher than those at 12 h. This consistent pattern suggested a time-dependent progression of liver injury. However, due to the limited sample size in our study (*n* = 3 per group), the Mann–Whitney U test was unable to detect statistical significance at the conventional α = 0.05 level (the minimum achievable exact *p* value for the Mann–Whitney U test was 0.1). Future investigations with larger sample sizes are warranted to validate whether these observed differences reach statistical significance.

Previous studies have indicated that triptolide, the major active component of TGE, induces oxidative stress and cellular damage in HepG2 cells [37]. SOD serves as a key free radical-scavenging enzyme that protects against oxidative stress-induced injury [38,39]. Both SOD and GSH-Px are established markers of antioxidant defense, while MDA—the end product of lipid peroxidation—serves as an important parameter reflecting the body’s antioxidant capacity [39,40,41]. In this study, TGE treatment resulted in decreased activities of SOD, GSH, and GSH-Px, accompanied by increased MDA levels. GSH levels were significantly reduced at 12 h, while MDA levels in the TGE-H group were significantly elevated at both 12 and 24 h. These changes indicated compromised hepatic antioxidant capacity, leading to MDA accumulation, enhanced lipid peroxidation, and aggravated liver injury. After 24 h, GSH concentration increased and MDA levels decreased in PCLSs from the TGE-L group, which may reflect a hepatocyte self-repair response stimulated by TGE. These results collectively indicate that TGE exposure intensified oxidative stress within PCLSs.

**Figure 9:**
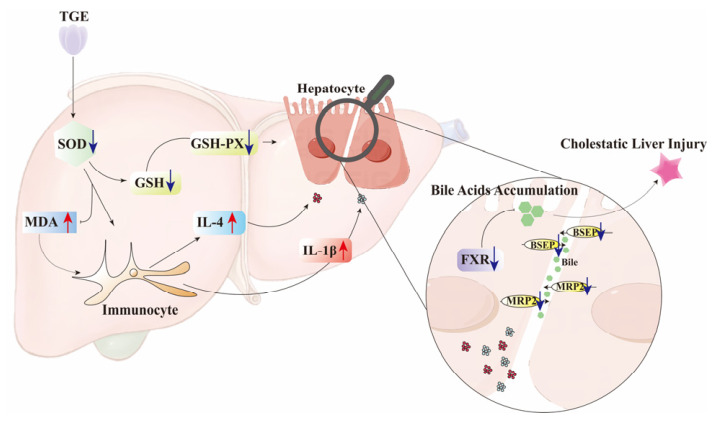
Schematic illustration of the proposed mechanism of TGE-induced cholestatic liver injury (created with Adobe Illustrator CS6, Adobe Systems Incorporated, San Jose, CA, USA.).

The intensification of oxidative stress can trigger the release of pro-inflammatory cytokines and activate various immune cells to release chemokines and cytokines, leading to immuno-inflammatory responses [42,43]. IL-1β, a potent pro-inflammatory cytokine, plays a crucial role in the host defense response to infection and injury [44]. Accumulating evidence has demonstrated that pro-inflammatory cytokines, including IL-1, IL-6, and monocyte chemoattractant protein-1 (MCP-1), play critical roles in the occurrence and development of acute liver failure, collectively contributing to hepatocyte death and liver failure [45,46]. It has been demonstrated that one aspect of triptolide-induced hepatotoxicity involves significantly increased IL-4 release, which also contributes to the development of cholestatic liver injury [10]. In the present study, we found that IL-1β (in all TGE-treated groups) and IL-4 (only in TGE-L group) levels were significantly elevated in TGE-treated groups at 24 h within the microfluidic liver-on-a-chip system. Although IL-4 elevation may promote TNF-α secretion, ultimately leading to hepatic injury [47,48], no significant difference in TNF-α levels was observed between the control and TGE-treated groups in our study. These cytokines activate membrane receptors on hepatocytes and cholangiocytes, transducing intracellular signals that result in altered expression and function of transporter proteins. IL-1β can downregulate mRNA expression levels of *BSEP*, *MRP2*, and *MRP3*, as well as mRNA and protein levels of FXR [49,50]. Studies in rodents have shown that increased IL-1β expression consistently reduces expression of the major bile acid transport proteins NTCP and BSEP, whereas TNF-α only transiently inhibits MRP2 expression [51,52]. In our study, MRP2 and BSEP expression levels decreased significantly in the TGE-treated groups after 24 h of culture, which may be attributable to increased IL-1β expression. Furthermore, in the presence of elevated inflammatory factors (IL-1β and NF-κB), the expression of FXR—a key regulator of bile acid homeostasis—is suppressed [53]. Alterations in FXR protein expression and function, along with changes in transporters responsible for bile acid efflux, can lead to intrahepatic bile acid retention and accumulation, ultimately resulting in cholestasis. The significant upregulation of IL-1β expression, coupled with significant decreases in BSEP and MRP2 protein levels, suggests that IL-1β may play a more prominent role in regulating BSEP and MRP2 expression. It should be noted that the measured levels of IL-1β and IL-4 indeed showed significant differences between 12 h and 24 h. Although our primary focus was on the differences between the control and TGE-treated groups at the same time points, this time-dependent effect still suggests that inflammation-related responses emerged in the liver slices as the culture time was extended. Further optimization of culture conditions to attenuate the baseline immune response of PCLSs, or the implementation of a more intensive time-course sampling, would help us better delineate the direct contribution of TGE to the immune response. Collectively, these findings indicate that elevated inflammatory factors inhibited expression of FXR and bile acid transporters such as BSEP and MRP2, thereby promoting bile acid accumulation within the liver and ultimately leading to hepatic cholestasis (Figure 9).

A major limitation of this study is the small sample size (*n* = 3, 4, or 5 biological replicates per group). With only three independent experiments, the *p*-values are sensitive to individual data points and may be unstable. Therefore, while our findings are statistically significant and consistently observed across three independent replicates, they should be interpreted as preliminary and hypothesis-generating. Nevertheless, the PCLS technique is an established in vitro model in which a sample size of *n* = 3–4 per group is widely accepted as the field standard [23,54], and similar sample sizes have been shown to be sufficient for detecting moderate to large effect sizes in microphysiological systems [55]. Importantly, their sample size analysis provides quantitative guidance that *n* = 3 is acceptable for pilot studies when effects are consistent across replicates. Future studies with larger sample sizes are warranted to validate and extend these findings.

## 4. Materials and Methods

### 4.1. Plant Material

TGE was purchased from Hunan Qianjin Xieli Pharmaceutical Co., Ltd. (Zhuzhou, China) in the form of tablets, each containing 10 mg of TGE (Lot No.:20230202). Twenty TGTs were determined to have a weight of 1.4152 g. The tablets were finely ground and transferred into a stoppered Erlenmeyer flask. Anhydrous ethanol (30 mL) was added, and the mixture was subjected to ultrasonication for 30 min (400 W, 40 kHz). After cooling, the mixture was filtered, and the resulting filtrate was evaporated to dryness. The mass of the dried filtrate was recorded as 121.90 mg, corresponding to an extraction yield of 8.60%. RP-HPLC analysis revealed that this TGE (121.90 mg) contained 0.4878 mg of wilforlide A, corresponding to a mass fraction of 0.400%, and 0.1194 mg of triptolide, corresponding to a mass fraction of 0.0979%.

### 4.2. Cell Culture

Human umbilical vein endothelial cells (EA.hy926; Resource No. FH0279) were obtained from Shanghai Fuheng Biology Co., Ltd. (Shanghai, China). The cells were cultured in DMEM supplemented with 10% fetal bovine serum (FBS; Gibco, Thermo Fisher Scientific Inc., Waltham, MA, USA), 100 U/mL of penicillin, and 0.1 mg/mL of streptomycin (P/S; Gibco, Thermo Fisher Scientific Inc., Waltham, MA, USA) and maintained at 37 °C in a humidified incubator with 5% CO_2_.

### 4.3. Reparation of Precision Cut Liver Slices

Five female specific-pathogen-free (SPF) Sprague-Dawley (SD) rats (120–180 g) were obtained from Beijing Vital River Laboratory Animal Technology Co., Ltd. (Beijing, China) [Animal Qualification Certificate No. SCXK (Jing) 2021-0011]. All experimental procedures were initiated following a 3-day acclimatization period after the animals arrived at the facility. The rats were housed under the following controlled environmental conditions: a temperature of 23 ± 3 °C, relative humidity of 40–70%, a 12 h light/dark cycle, and 15 air changes per hour. The animals were provided with filtered tap water and pelleted rat chow ad libitum.

General anesthesia was induced in all animals via intramuscular injection of Zoletil^®^ 50 (40 mg/kg; Virbac SA, Carros, France). Under sterile conditions, rat livers were excised, washed three times with pre-cooled DMEM, and subsequently placed in DMEM that had been pre-saturated and continuously supplied with oxygen (95% O_2_; 5% CO_2_). Precision-cut liver slices (PCLSs) with a thickness of 300 µm and a diameter of 6 mm were then prepared [56].

### 4.4. Cytotoxicity Detection

EA.hy926 cells were seeded in 96-well plates at a density of 1 × 10^4^ cells per well and cultured in a cell culture incubator at 37 °C with 5% CO_2_ for 24 h prior to treatment. The cells were then exposed to varying concentrations of TGE: 30.03, 40.05, 53.39, 71.19, 94.92, 125.56, 168.75, 225.00, and 300.00 μg/mL. Following a 24 h incubation period, the supernatant was removed, and 110 μL of CCK-8 solution (DOJINDO Laboratories, Kumamoto, Japan) was added to each well. After further incubation at 37 °C for 30 min, absorbance (A) was measured at 450 nm. Cell viability (%) and the half-maximal inhibitory concentration (IC_50_) of TGE on EA.hy926 cells were subsequently calculated.

HepG2 cells were seeded in 96-well plates at a density of 1 × 10^4^ cells per well and cultured in a cell culture incubator at 37 °C with 5% CO_2_ for 24 h prior to treatment. The cells were then exposed to varying concentrations of TGE: 6.25, 12.50, 25.00, 50.00, 100.00, 150.00, and 200.00 μg/mL. Following a 24 h incubation period, the supernatant was removed, and 110 μL of CCK-8 solution (DOJINDO, Japan) was added to each well. After further incubation at 37 °C for 30 min, absorbance (A) was measured at 450 nm. The inhibition rate (%) and the half-maximal inhibitory concentration (IC_50_) of TGE on HepG2 cells were subsequently calculated. Based on these results, concentrations corresponding to IC_5_, IC_15_, and IC_45_ were selected for subsequent experiments, with the additional criterion that these concentrations exhibited no cytotoxic effects on EA.hy926 cells.

### 4.5. Microfluidic Liver -on-a-Chip System

**Figure 10:**
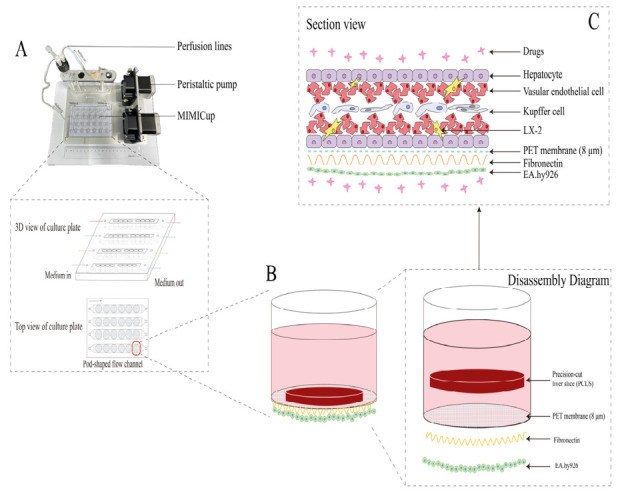
Physical image of microfluidic liver organ-on-a-chip system and schematic diagram of its internal structure. (**A**) Physical image of microfluidic liver organ-on-a-chip system (the picture above), and 3D view of culture plate and top view of culture plate (the picture below). (**B**) Structure of culture plate MIMICup and layered arrangement of cells and PCLS. (**C**) Schematic diagram of biomimetic liver lobule structure (created with Adobe Illustrator CS6, Adobe Systems Incorporated, San Jose, CA, USA.).

The microfluidic liver-on-a-chip system utilized in this study was procured from Shanghai Aurefluidics Technology Co., Ltd. (Shanghai, China). The architecture of the chip is depicted in Figure 10. The device comprised three distinct layers and four integrated channels. The uppermost layer consisted of a polycarbonate (PC) MIMICup culture chamber, the base of which was fitted with a polyethylene terephthalate (PET) membrane. PCLSs were cultured on the upper surface of this membrane. The lower surface of the PET membrane formed the second layer of the chip, onto which EA.hy926 cells were seeded at a density of 2.72 × 10^5^ cells/mL with an inoculation volume of 100 μL per well to serve as a surrogate for sinusoidal endothelial cells, as previously described [57]. The third layer featured a pod-shaped microchannel architecture, constructed from a polycarbonate (PC) runner plate and sealed with a polymethyl methacrylate (PMMA) film. This pod-like channel was designed to recapitulate the structural characteristics of the hepatic sinusoid. Each pod-shaped channel accommodated six individual MIMICup culture chambers. Following the assembly of all structural components, the system was integrated with a micro-peristaltic pump and controller via a perfusion line to establish a fully functional chip platform. We have previously demonstrated the physiological validity of the liver-on-a-chip system using techniques including H&E staining, co-incubation with specific metabolic probes, and immunofluorescence staining of tight junction proteins. These validations confirmed the viability of PCLS, the enzymatic activity of CYP450 (CYP3A1/2, CYP2E1), and the dense endothelial barrier established by EA.hy926 cells via tight junctions [58].

Before activating the controller, all components of the microfluidic liver-on-a-chip perfusion system were connected and properly sealed. DMEM culture medium containing 0.5% (*v*/*v*) DMSO and supplemented with TGE at concentrations of 15.00, 45.00, and 135.00 μg/mL was used as the circulating fluid. These concentrations were selected based on the results of the cytotoxicity assays and were designated as the TGE-L, TGE-M, and TGE-H groups, respectively. Each MIMICup was loaded with 180 μL of the corresponding drug-containing medium. For the control group (CON group), both the circulating fluid and the medium within the MIMICups consisted of DMEM supplemented with 0.5% (*v*/*v*) DMSO alone. The flow rate of the micro-peristaltic pump was subsequently adjusted to 251 μL/min. The entire system was then maintained in an incubator at 37 °C with 5% CO_2_ for either 12 or 24 h.

### 4.6. Sampling

Following drug administration and subsequent culture for either 12 or 24 h, the circulating fluid (representing the bionic blood) was collected from the microfluidic liver-on-a-chip system for biochemical analysis. The MIMICups were then detached from the system. EA.hy926 cells adherent to the lower surface of the PET membrane were fixed with 4% neutral formaldehyde. From each experimental group, a subset of EA.hy926 cells within the MIMICups was subjected to hematoxylin and eosin (H&E) staining, while the remaining cells were processed for immunofluorescence (IF) staining. PCLSs from each group were also retrieved from the MIMICups. PCLSs from one rat were fixed with 4% neutral formaldehyde for morphological evaluation. PCLSs from a second rat were fixed with 4% neutral formaldehyde for immunohistochemical (IHC) analysis. PCLSs from a third rat were collected to determine the malondialdehyde (MDA) content and the activities of superoxide dismutase (SOD), reduced glutathione (GSH), and glutathione peroxidase (GSH-Px); PCLSs from a fourth rat were used for subsequent enzyme-linked immunosorbent assay (ELISA); and PCLSs from a fifth rat were used for Western blot analysis. The experiment unit was PCLS.

### 4.7. Morphological Observation and Immunofluorescence Staining of EA.hy926 Cells

Following fixation, EA.hy926 cells from each experimental group were washed three times with PBS buffer. Subsequently, 0.3% Triton X-100 was applied to the PET membrane and incubated at room temperature for 15 min. The cells were then washed three times with PBS, after which 1% BSA was added for blocking and incubated at room temperature for 30 min. Following the removal of the blocking solution, cells were incubated with primary antibodies against ZO-1 (Cat No. 21773-1-AP; Proteintech Group, Inc., Wuhan, China) or VE-cadherin (Cat No. 2158S; Cell Signaling Technology, Inc., Danvers, MA, USA) overnight at 4 °C. After three additional washes with PBS, the cells were incubated with FITC-conjugated goat anti-rabbit IgG (H+L) secondary antibody (Cat No. SA00003-2; Proteintech, China) for 1 h at room temperature in the dark. Following another round of PBS washes, nuclei were counterstained with Hoechst 33342 for 10 min in the dark. After three final washes with PBS, the PET membrane was carefully detached from the MIMICup and placed cell-side up onto a glass slide. The sample was then mounted with 50% glycerol and observed under an ECLIPSE Ni-U fluorescence microscope (NIKON CORPORATION, Tokyo, Japan). Each group had 3 to 4 PET membranes, and 1 random field of view from each PET membrane was captured. The fluorescence intensity of VE-cadherin or ZO-1 was quantified using the Image J software Fiji (National Institutes of Health, Bethesda, MD, USA). The mean fluorescence intensity was measured in each field, and the average of three or four fields per group was used for statistical analysis. All image acquisitions and quantifications were performed blinded to the experimental groups.

### 4.8. Bionic Blood Biochemical Detection

The levels of biomarkers associated with liver cell damage, including AST, mitochondrial AST (mAST), ALT, and lactate dehydrogenase (LDH); the bile duct damage biomarker γ-glutamyl transferase (GGT); and markers of liver and gallbladder excretory function, namely direct bilirubin (DBIL) and total bile acid (TBA), were measured in the circulating bionic blood collected from each microfluidic liver-on-a-chip system. All analyses were performed using a TBA-40 FR Automatic Biochemical Analyzer (Toshiba Medical Systems Corporation, Otawara, Tochigi, Japan).

### 4.9. Morphological Observation of PCLSs

PCLSs from each experimental group were dehydrated, cleared, and embedded in paraffin using a VP1-JC automated tissue processor (Sakura Seiki Co., Ltd., Chikuma, Japan) and a Tissue-Tek^®^ TEC^™^5 tissue embedding console (Sakura, Japan). Sections with 4 μm thickness were subsequently prepared and stained with hematoxylin and eosin for histopathological examination under a Ni-U microscope (Nikon, Japan).

Histological sections were assessed blindly. Semi-quantitative scoring of PCLSs was conducted using the scoring system described in Table 3. Four histopathological parameters (hepatocyte swelling/degeneration, edema, sinusoidal dilation, and karyolysis) were assessed, and each parameter was graded on a scale from 0 to 3 (0 = none, 1 = mild/focal, 2 = moderate/multifocal, and 3 = severe/diffuse). The total lesion score was calculated for each sample by summing the individual parameter scores, yielding a result in the range of 0 to 12.

### 4.10. Malonaldehyde, Glutathione, Glutathione Peroxidase and Superoxide Dismutase Levels in PCLSs in the MIMICups in the Microfluidic Liver-on-a-Chip System

Following drug administration and subsequent culture for 12 and 24 h, PCLSs were harvested and homogenized. The homogenates were centrifuged at 3000 rpm for 10 min, and the resulting supernatants were collected to determine the malondialdehyde (MDA), reduced glutathione (GSH), glutathione peroxidase (GSH-Px), and superoxide dismutase (SOD) levels. MDA concentrations were measured using an MDA assay kit based on the thiobarbituric acid method (Nanjing Jiancheng Bioengineering Institute, Nanjing, China). The GSH content was assessed using a reduced glutathione assay kit (Nanjing Jiancheng Bioengineering Institute, China). GSH-Px activity was evaluated with a glutathione peroxidase assay kit employing a colorimetric method (Nanjing Jiancheng Bioengineering Institute, China). SOD activity in hepatocytes was determined using a total superoxide dismutase (T-SOD) assay kit based on the hydroxylamine method (Nanjing Jiancheng Bioengineering Institute, China).

### 4.11. ELISA

The levels of tumor necrosis factor alpha (TNF-α), interleukin-1β (IL-1β), and interleukin-4 (IL-4) in the supernatants obtained from PCLS homogenates were quantified using ELISA kits according to the manufacturers’ instructions. Specifically, TNF-α was measured with the BD OptEIA^™^ Rat TNF ELISA kit (Cat. No. 560479; BD, Franklin Lakes, NJ, USA), IL-1β with the Rat IL-1β ELISA Kit (Cat. No. CSB-E08055r-IS; CUSABIO, Wuhan, Hubei, China), and IL-4 with the BD OptEIA^™^ Rat IL-4 ELISA Set (Cat. No. 555198; BD, USA).

### 4.12. Western Blot Analysis

Fresh frozen PCLSs from each experimental group were homogenized in lysis buffer. Protein electrophoresis and electrotransfer were performed as described in previous studies [59,60]. The PVDF membranes were blocked by incubation in 5% (*w*/*v*) BSA in PBS containing Tween-20 for 2 h at room temperature, followed by overnight incubation at 4 °C with primary antibodies against farnesoid X receptor (FXR; mouse anti-FXR antibody, 1:5000, Cat. No. 67813-1-Ig; Proteintech, China), multidrug resistance-associated protein 2 (MRP2; rabbit anti-MRP2 antibody, 1:100, Cat. No. bs-1092R; BIOSS, Beijing, China), and β-actin (rabbit anti-β-actin antibody, 1:2000, Cat. No. 81115-1-RR; Proteintech, China). After three washes with PBS containing Tween-20, the membranes were incubated for 1 h at room temperature with horseradish peroxidase-conjugated goat anti-rabbit or anti-mouse IgG secondary antibodies (1:2000, Cat. No. SA00001-2 and SA00001-1; Proteintech, China). Immunoreactive bands were visualized using the Immobilon^®^ Western Chemiluminescent HRP Substrate Detection Kit (Cat. No. WBKLS0100; Merck Millipore, Burlington, MA, USA) and imaged with an Amersham^™^ Imager 680 system (GE Healthcare Life Sciences, Marlborough, MA, USA). Densitometric analysis was subsequently performed. Western blot bands were scanned and quantified using Image J. The signal intensity of each target protein was normalized to the corresponding loading control (β-actin). Three PCLSs per group were subjected to Western blot analysis. The density of each protein band was measured using Image J, with each band quantified once. Blinding was applied during band selection and densitometric measurement.

### 4.13. Immunohistochemical (IHC) Staining

Sections of PCLSs with a thickness of 4 μm were prepared for IHC analysis. IHC staining was performed in accordance with the manufacturer’s instructions using a two-step detection kit for mouse primary antibodies (Mouse Enhanced Polymer Method Detection System, PV-9002; ZSGBBIO, Beijing, China). Mouse anti-Bile Salt Export Pump (BSEP) antibody (1:5000, Cat. No. 3C11D5; Proteintech, China) was used as the primary antibody. Positive immunoreactivity for BSEP was identified as granular yellow or brown deposits within hepatocytes. BSEP immunostaining intensity was semi-quantitatively assessed using the Image J software. For each group, five random fields were captured. The integrated optical density (IOD) of BSEP was measured after background correction. All analyses were performed blinded to the experimental groups.

### 4.14. Statistical Analysis

Statistical analysis was performed using GraphPad Prism 8.0 (GraphPad Software, Inc., San Diego, CA, USA). All data (excluding the histological semi-quantitative scoring data) are expressed as the mean ± standard deviation (SD). For each time point, normality of residuals was assessed using the Shapiro–Wilk test. Homogeneity of variances was assessed using Levene’s test. When normality and homoscedasticity assumptions were met (*p* > 0.05 for both tests), a one-way analysis of variance (ANOVA) was performed to compare the four groups (CON, TGE-L, TGE-M, and TGE-H). Following a significant ANOVA, Dunnett’s multiple comparisons test was applied to compare each TGE treatment group with the common control group. When normality or homoscedasticity assumptions were violated (*p* < 0.05 for either test), the Kruskal–Wallis test was used for overall group comparison, followed by Dunn’s post hoc test with Bonferroni correction for pairwise comparisons between each treatment group and the control group. A significance threshold of α = 0.05 was used for all comparisons. *P*-values < 0.05 were considered statistically significant.

Histological score data are ordinal in nature. Therefore, non-parametric tests were employed. Data are presented as medians (range). (a) Comparisons among multiple independent groups at the same time point were performed using the Kruskal–Wallis test, followed by Dunn’s multiple comparisons test for post hoc pairwise comparisons between treatment groups and the control group at the same time point. A *p*-value < 0.05 was considered statistically significant. (b) For comparisons between the same treatment group at different time points (e.g., TGE-L group at 12 h vs. 24 h), the Mann–Whitney U test was employed, as the data were ordinal and did not follow a normal distribution, and the PCLSs at the two time points were independent. All comparisons were two-tailed, and a *p* value < 0.05 was considered statistically significant. All analyses were performed using GraphPad Prism 8.0 (GraphPad Software, Inc., San Diego, CA, USA).

## 5. Conclusions

TGE triggered an immune response in hepatocytes by stimulating oxidative stress and inducing lipid peroxidation in rat PCLSs, thereby accelerating hepatocyte injury and ultimately leading to cholestasis. This process was associated with the inhibition of FXR, MRP2, and BSEP expression, as well as elevated levels of IL-1β across all TGE-treated groups and IL-4 in the TGE-L group at the 24 h time point. In this study, we successfully recapitulated TGE-induced cholestatic liver injury at the microscale using a microfluidic liver-on-a-chip platform and further demonstrated the feasibility of this technology for the evaluation and mechanistic investigation of drug-induced liver injury (DILI).

## Figures and Tables

**Table 1:** Effect of TGE on the levels of liver injury markers in circulating bionic blood in microfluidic liver organ-on-a-chip system (*n* = 3).

Time	Group	Concentration of TGE (μg/mL)	AST (U/L)	mAST (U/L)	ALT (U/L)	LDH (U/L)	GGT (U/L)	TBA (μmol/L)	DBIL (μmol/L)
12 h	CON	0	57.17 ± 2.70	26.48 ± 2.76	5.85 ± 1.60	177.80 ± 32.92	2.25 ± 0.08	1.13 ± 0.02	0.33 ± 0.12
	TGE-L	15	62.93 ± 8.73	28.79 ± 8.98	5.99± 1.06	203.79 ± 61.13	2.30 ± 0.04	1.31 ± 0.21	0.29 ± 0.11
	TGE-M	45	78.63 ± 37.24	46.60 ± 35.77	8.24 ± 1.75	441.67 ± 210.00	2.23 ± 0.18	1.22 ± 0.19	0.31 ± 0.10
	TGE-H	135	69.77 ± 10.68	36.80 ± 9.39	7.44 ± 0.74	276.95 ± 60.08	2.25 ± 0.09	1.45 ± 0.09	0.33 ± 0.06
24 h	CON	0	75.00 ± 20.22	51.87 ±16.22	5.62 ± 2.98	176. 40 ± 1.73	2.03 ± 0.16	3.30 ± 0.20	0.47 ± 0.02
	TGE-L	15	65.67 ± 32.39	46.77 ± 24.03	10.45 ± 3.46	228.77 ± 134.01	1.99 ± 0.51	3.87 ± 0.25	0.57 ± 0.09
	TGE-M	45	139.33 ± 0.58 *	109.63 ± 1.36 **	10.18 ± 1.74	541.73 ± 3.50 ***	2.41 ± 0.22	4.30 ± 0.35 *	0.57 ± 0.04
	TGE-H	135	72.33 ± 19.66	52.83 ± 14.90	12.83 ± 2.81 *	264.10 ± 16.84	2.62 ± 0.27	4.03 ± 0.67	0.66 ± 0.05 **

Note: Data are means ± SD. * *p* < 0.05, ** *p* < 0.01, *** *p* < 0.001 compared with the control group.

**Table 2:** Semi-quantitative histological scoring of PCLSs following TGE treatment.

Group	Time (h)	Hepatocyte Swelling/Degeneration	Edema	Sinusoidal Dilation	Karyolysis	Total Score (Median, Range)
CON	12	0 (0–0)	0 (0–0)	0 (0–0)	0 (0–0)	0 (0–0)
TGE-L	12	1 (0–1)	0 (0–0)	0 (0–0)	0 (0–0)	1 (0–1)
TGE-M	12	1 (1–2)	0 (0–0)	0 (0–0)	0 (0–1)	1 (1–2)
TGE-H	12	2 (2–2)	0 (0–1)	0 (0–1)	1 (1–1)	3 (3–4) *
CON	24	0 (0–0)	0 (0–0)	1 (1–2)	0 (0–0)	1 (1–2)
TGE-L	24	2 (2–2)	2 (1–2)	2 (1–2)	0 (0–0)	5 (4–6)
TGE-M	24	2 (2–2)	1 (0–1)	1 (1–2)	2 (2–2)	6 (5–7)
TGE-H	24	3 (2–3)	2 (1–2)	2 (2–3)	3 (2–3)	9 (9–10) *

Note: Data are presented as median (range), *n* = 3 per group. Culture for 12 h: TGE-L vs. CON, *p* > 0.9999; TGE-M vs. CON, *p* = 0.5285; TGE-H vs. CON, *p* = 0.0162. Culture for 24 h: TGE-L vs. CON, *p* > 0.9999; TGE-M vs. CON, *p* = 0.4085; TGE-H vs. CON, *p* = 0.0125. * *p* < 0.05 compared with the control group. For comparisons of the same treatment group between two time points (12 h vs. 24 h), the Mann–Whitney U test was used. Due to the small sample size (*n* = 3 per group), the minimum achievable exact *p* value for the Mann–Whitney U test was 0.1.

**Table 3:** Semi-quantitative histological scoring system for PCLSs in microfluidic liver organ-on-a-chip system.

Score	Hepatocyte Swelling/Degeneration	Edema	Sinusoidal Dilation	Karyolysis
0	None	None	None	None
1	Mild, focal	Mild, focal	Mild, focal	Occasional, focal
2	Moderate, multifocal	Moderate, multifocal	Moderate, multifocal	Moderate, multifocal
3	Severe, diffuse	Severe, diffuse	Severe, diffuse	Severe, diffuse (prominent)

Note: Total lesion score = sum of four parameter scores (range: 0–12).

## Data Availability

The original contributions presented in this study are included in the article/Supplementary Materials. Further inquiries can be directed to the corresponding author.

## Supplementary Materials

The following supporting information can be downloaded at https://www.mdpi.com/article/10.3390/ijms27094154/s1.

## Abbreviations

The following abbreviations are used in this manuscript:

TGE	Tripterygium Glycosides Extraction
PCLSs	Precise Cut Liver Slices
FXR	Farnesoid X Receptor
MRP2/3	Multi-drug Resistance Protein 2/3
BSEP	Bile Salt Export Pump
TGT	Tripterygium Glycosides Tablet
ADR	Adverse Drug Reaction
ALT	Alanine Aminotransferase
AST	Aspartate Aminotransferase
mAST	Mitochondrial Aspartate Aminotransferase
ALP	Alkaline Phosphatase
TP	Triptolide
DILI	Drug-Induced Liver Injury
MCP-1	Monocyte Chemoattractant Protein-1
SOD	Superoxide Dismutase
MDA	Malondialdehyde
GSH	Glutathione
GSH-Px	Glutathione Peroxidase
TNF-α	Tumor Necrosis Factor-alpha
IL-1/6	Interleukin-1/6
TBA	Total Bile Acid
GGT	γ-glutamyl Transferase
DBIL	Direct Bilirubin
TBIL	Total Bilirubin
LDH	Lactate Dehydrogenase
