# Supplementary material for: Tripterygium Glycosides Extract-Induced Hepatic Cholestasis: A Mechanistic Study Using a Microfluidic Liver-on-a-Chip System
Source: Int J Mol Sci. 2026 May 6;27(9):4154. doi: 10.3390/ijms27094154 (PMC13164438; doi:10.3390/ijms27094154)
Supplement: Supplementary file 1 [file ijms-27-04154-s001.zip › Supplementary Files Table S1 individual data of histological scoring.docx]

Table S1. Individual animal semi-quantitative histological scores for all experimental groups.

| Group | Animal ID | Hepatocyte swelling/degeneration | Edema | Sinusoidal dilation | Karyolysis | Total score |
| --- | --- | --- | --- | --- | --- | --- |
| CON(12h) | C1 | 0 | 0 | 0 | 0 | 0 |
|  | C2 | 0 | 0 | 0 | 0 | 0 |
|  | C3 | 0 | 0 | 0 | 0 | 0 |
| TGE-L (12h) | L1 | 1 | 0 | 0 | 0 | 1 |
|  | L2 | 1 | 0 | 0 | 0 | 1 |
|  | L3 | 0 | 0 | 0 | 0 | 0 |
| TGE-M (12h) | M1 | 1 | 0 | 0 | 0 | 1 |
|  | M2 | 1 | 0 | 0 | 0 | 1 |
|  | M3 | 2 | 0 | 0 | 0 | 2 |
| TGE-H (12h) | H1 | 2 | 0 | 0 | 1 | 3 |
|  | H2 | 2 | 0 | 0 | 1 | 3 |
|  | H3 | 2 | 0 | 1 | 1 | 4 |
| CON (24h) | C4 | 0 | 0 | 1 | 0 | 1 |
|  | C5 | 0 | 0 | 1 | 0 | 1 |
|  | C6 | 0 | 0 | 2 | 0 | 2 |
| TGE-L (24h) | L4 | 2 | 2 | 1 | 0 | 4 |
|  | L5 | 2 | 2 | 2 | 0 | 6 |
|  | L6 | 2 | 1 | 2 | 0 | 5 |
| TGE-M (24h) | M4 | 2 | 1 | 1 | 2 | 6 |
|  | M5 | 2 | 1 | 2 | 2 | 7 |
|  | M6 | 2 | 0 | 1 | 2 | 5 |
| TGE-H (24h) | H4 | 3 | 2 | 2 | 3 | 10 |
|  | H5 | 2 | 2 | 3 | 2 | 9 |
|  | H6 | 3 | 1 | 2 | 3 | 9 |
